# The Metabolic Fate of Deoxynivalenol and Its Acetylated Derivatives in a Wheat Suspension Culture: Identification and Detection of DON-15-*O*-Glucoside, 15-Acetyl-DON-3-*O*-Glucoside and 15-Acetyl-DON-3-Sulfate

**DOI:** 10.3390/toxins7083112

**Published:** 2015-08-12

**Authors:** Clemens Schmeitzl, Benedikt Warth, Philipp Fruhmann, Herbert Michlmayr, Alexandra Malachová, Franz Berthiller, Rainer Schuhmacher, Rudolf Krska, Gerhard Adam

**Affiliations:** 1 Department of Applied Genetics and Cell Biology, University of Natural Resources and Life Sciences, Vienna (BOKU), Konrad-Lorenz-Straße 24, 3430 Tulln, Austria; E-Mails: herbert.michlmayr@boku.ac.at (H.M.); gerhard.adam@boku.ac.at (G.A.); 2 Center for Analytical Chemistry, Department for Agrobiotechnology (IFA-Tulln), University of Natural Resources and Life Sciences, Vienna (BOKU) Konrad-Lorenz-Straße 20, 3430 Tulln, Austria; E-Mails: benedikt.warth@univie.ac.at (B.W.); alexandra.malachova@boku.ac.at (A.M.); franz.berthiller@boku.ac.at (F.B.); rainer.schuhmacher@boku.ac.at (R.S.); rudolf.krska@boku.ac.at (R.K.); 3 Institute of Applied Synthetic Chemistry, Vienna University of Technology, Getreidemarkt 9/163-OC, 1060 Vienna, Austria; E-Mail: philipp.fruhmann@tuwien.ac.at; 4 Christian Doppler Laboratory for Mycotoxin Metabolism, Konrad-Lorenz-Straße 20, 3430 Tulln, Austria

**Keywords:** carboxylesterase, *Fusarium*, 3-acetyl-deoxynivalenol, 15-acetyl-deoxynivalenol, 3,15-diacetyl-deoxynivalenol, masked mycotoxins, chemotype

## Abstract

Deoxynivalenol (DON) is a protein synthesis inhibitor produced by the *Fusarium* species, which frequently contaminates grains used for human or animal consumption. We treated a wheat suspension culture with DON or one of its acetylated derivatives, 3-acetyl-DON (3-ADON), 15-acetyl-DON (15-ADON) and 3,15-diacetyl-DON (3,15-diADON), and monitored the metabolization over a course of 96 h. Supernatant and cell extract samples were analyzed using a tailored LC-MS/MS method for the quantification of DON metabolites. We report the formation of tentatively identified DON-15-*O*-β-D-glucoside (D15G) and of 15-acetyl-DON-3-sulfate (15-ADON3S) as novel deoxynivalenol metabolites in wheat. Furthermore, we found that the recently identified 15-acetyl-DON-3-*O*-β-D-glucoside (15-ADON3G) is the major metabolite produced after 15-ADON challenge. 3-ADON treatment led to a higher intracellular content of toxic metabolites after six hours compared to all other treatments. 3-ADON was exclusively metabolized into DON before phase II reactions occurred. In contrast, we found that 15-ADON was directly converted into 15-ADON3G and 15-ADON3S in addition to metabolization into deoxynivalenol-3-*O*-β-D-glucoside (D3G). This study highlights significant differences in the metabolization of DON and its acetylated derivatives.

## 1. Introduction

Deoxynivalenol (DON) is a mycotoxin produced by various members of the *Fusarium graminearum* species complex causing Fusarium head blight on different crop species. DON belongs to the structurally diverse group of trichothecenes which is characterized by a tricyclic 12,13-epoxytrichothec-9-ene structure [1]. Trichothecenes contain an epoxide at C-12 and C-13, which is essential for its toxicity, based on inhibition of eukaryotic protein synthesis [2]. Uptake of DON causes acute and chronic symptoms including emesis, abdominal distress, growth retardation and immune dysregulation [3,4,5,6]. The European Food Safety Authority (EFSA) reviewed the occurrence of DON in food and feed samples obtained in the European Union between 2007 and 2012. About 43.5% of the grain samples for human consumption were positive for DON and 35.7% were above 100 µg/kg with an average of 112 µg/kg [7]. The average contribution of acetylated DON derivatives to the total DON value was found to be up to 20% [7]. The American Association of Cereal Chemists (AACC) reported that between 2003 and 2014 18.9% of all wheat samples shipped to milling facilities contained at least 300 to 500 µg/kg with an calculated average of 850 µg/kg for 2014 [8]. For soft wheat, as much as 30% of all samples contained above 2000 µg/kg.

The most prevalent Fusarium head blight pathogens for wheat are *Fusarium graminearum* and *F. asiaticum,* which has a high importance especially in Asia [9]. Trichothecene producing *Fusarium* strains can be grouped into chemotypes, based on the main metabolite produced in axenic cultures: nivalenol (NIV) producer, deoxynivalenol (DON) producer and the recently discovered NX producers [10,11]. NX-3 (7α,15-dihydroxy-12,13-epoxytrichothec-9-ene) and its acetylated derivate NX-2 are type A trichothecenes similar to DON but lacking the C-8 keto-group. Genetic analysis revealed that a mutated *TRI1* no longer oxidized calonectrin at C-8 but still at C-7 [10]. In contrast, both DON and NIV as well as their acetylated derivatives fusarenon X (4-acetyl-NIV), 3-acetyl-DON (3-ADON) and 15-acetyl-DON (15-ADON) belong to the type B trichothecenes due to their keto group at C-8. The DON chemotype strains can be further sub‑grouped into 3-ADON and 15-ADON strains. Depending on the allele of the carboxylesterase Tri8, either the C-15 or C-3 acetyl group is removed from the common biosynthetic precursor 3,15‑di‑acetyl-DON (3,15-diADON) [12]. The second deacetylation step to DON by the fungus occurs very slowly [12], so that during the infection process the plant is most likely, depending on the pathogen chemotype, challenged primarily with either 15-ADON or 3-ADON.

Trichothecenes are known for being potent protein synthesis inhibitors. Toxicity assessment via rabbit reticulocyte lysate and wheat germ based translation inhibition assay identified an IC_50_ of about 1.5 μM for both DON and 15-ADON [10], but in the case of 3-ADON, the IC_50_ is at least 100 times higher [13]. Acetylation at C-3 is regarded as a self-protection mechanism of the fungi, and 3,15-diADON is expected to have a comparable low affinity to the ribosome like 3-ADON [13]. As binding to the primary ribosomal target is expected to be similar in every cell type, discrepancies in various studies concerning toxicity could be explained by a combination of two effects: (i) depending on the model and the route of administration ADONs become deacetylated to a variable extent and (ii) acetylation affects the cellular uptake or the specific transport (by efflux carriers). For example, oral administration of 3-ADON in pigs results in the presence of DON and DON-glucuronide in the blood, but no 3-ADON was detected [14]. It was suggested that ADONs are deacetylated by intestinal lipases and/or epithelial carboxylesterases in monogastric species like pigs and humans [15]. DON and its acetylated derivatives are phytotoxic, but considering the results from the translation inhibition assay it is obvious that in the case of 3-ADON the phytotoxic effect [10,16] observed is due to deacetylation to DON. This is supported by the finding that over-expression of the acetyltransferase Tri101 in rice leads to resistance against DON due to the acetylation of DON to 3-ADON [17,18]. Little is known about genes in crop plant species affected by Fusarium head blight responsible for deacetylation.

In general, detoxification of the Fusarium virulence factor DON and its acetylated derivatives is achieved in three phases. In phase I xenobiotics are oxidized or hydrolyzed by esterases, amidases and the cytochrome P-450 system with the main aim to create reactive sites [19] including deacetylation of acetylated DON derivates. Phase II reactions are characterized by conjugation, resulting in less toxic or non-toxic metabolites [19]. The most prominent phase II metabolite of DON is deoxynivalenol-3-*O*-β-D-glucoside (D3G), but also the formation of DON-3-sulfate (D3S), DON-15-sulfate (D15S), DON-glutathione (DON-GSH), DON-malonylglucoside and DON-di-hexoside has been observed in plants [20,21,22,23,24]. Finally, phase III reactions in plants involve sequestration of the conjugated compounds into the vacuole or irreversible binding to the cell wall [25].

The objective of this work was to study the metabolic fate of DON and its acetylated derivatives utilizing a wheat suspension culture. We present experimental evidence for formation of three novel DON conjugates, DON-15-*O*-β-D-glucoside (D15G), 15-acetyl-DON-3-*O*-β-D-glucoside (15-ADON3G) and 15-acetyl-DON-3-sulfate (15-ADON3S) and highlight differences in the metabolization of DON and its acetylated derivatives.

## 2. Results and Discussion

**Figure 1:**
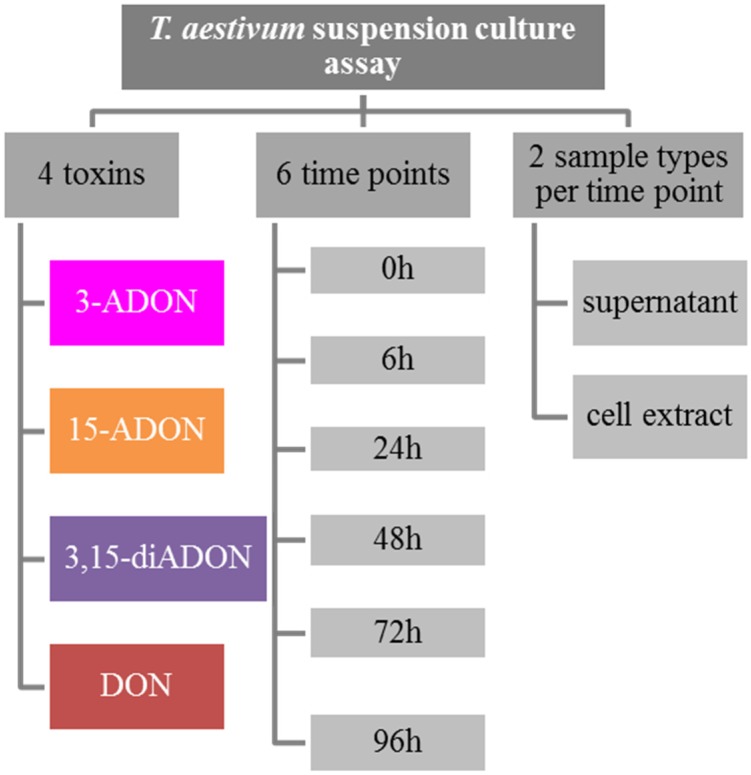
Experimental design of the wheat suspension culture experiment.

We monitored the metabolic fate of DON, 3-ADON, 15-ADON and 3,15-diADON in a *Triticum aestivum* suspension culture. A dense culture in fresh medium was supplemented separately with each of the four toxins and wheat cells as well as the supernatant and was sampled over a course of 96 h. The outline of the experiment is shown in Figure 1. For analysis of metabolites, an LC-MS/MS method was extended and employed as described in the Materials and Methods section.

### 2.1. Enzymatic Synthesis of 15-Acetyl-DON-3-O-d-Glucoside

**Figure 2:**
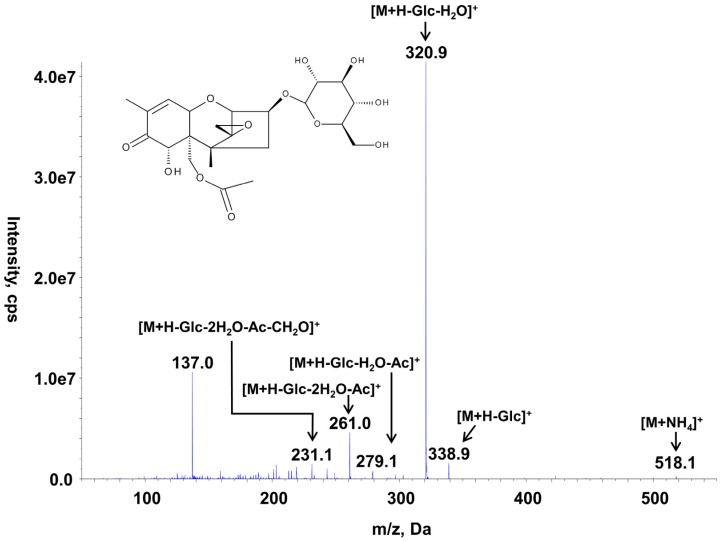
Chemical structure and MS/MS spectrum of 15-ADON-3-*O*-d-glucoside recorded at a collision energy of +20 eV. *m*/*z*: Mass-to-charge ratio, M: intact molecule, Glc: glucosyl, Ac: acetyl.

To prepare an analytical standard which allows quantification of 15-ADON3G, 15-ADON was enzymatically conjugated with UDP-glucose by utilizing a heterologously expressed and affinity purified UDP-glucosyltransferase from *Oryza sativa* (NM_001058779.1) [26]. One hundred microliters of a 10 mg/L 15-ADON solution was converted within two hours into 15-ADON3G with a yield of nearly 100%. Thereafter, the solution was diluted with pure ACN to a final ACN concentration of 50%. To compare the MS response of the synthesized 15-ADON3G relative to that of 15-ADON, the corresponding reference standards containing equimolar amounts of 15-ADON and 15-ADON3G (7.4 µM) were injected each three times and the corresponding peak areas were compared. The transition *m*/*z* 518→339 of the 15-ADON3G conjugate, which was the most abundant product ion at optimized collision energies (see Table 1), showed an approximately 44 times more abundant response than the transition *m*/*z* 339→261, which was used for the quantification of 15-ADON. In subsequent measurements, 15-ADON3G was quantified using the calibration of 15-ADON and the results corrected by the determined factor 44 since 15-ADON3G is quite unstable in water containing solvents (see below). In Figure 2, the MS/MS spectrum and the chemical structure of 15-ADON3G are displayed.

Further attempts to structurally confirm the 15-ADON3G standard by NMR failed due to its limited stability, especially in solvents with higher water content. Subsequently, we evaluated the stability of the above mentioned enzymatically produced standard directly after synthesis and addition of organic solvent (ACN) utilizing the described LC-MS/MS method. The standard was measured one, three and 24 h after synthesis. While the standard was stable during the first two measurements, a decrease in the concentration (*ca.* 90% remaining) was found after 24 h. The 10% missing was converted to D3G while no free 15-ADON or DON was detected. Besides this stability testing of a freshly prepared standard, we also investigated the fate of a nine month old standard. This standard was purified by preparative HPLC and initially contained 100 mg/L 15-ADON3G. After the NMR measurement, complete degradation was observed. Also, after nine months of storage at 4 °C, the purified 15-ADON3G was converted to either D3G (99.7%) or DON (*ca.* 0.2%). Only about 0.1% of 15-ADON3G was found to be in its original state. This confirms the instability of this conjugate and suggests that immediate sample analysis is warranted.

### 2.2. Treatment of the Suspension Culture with DON

**Figure 3:**
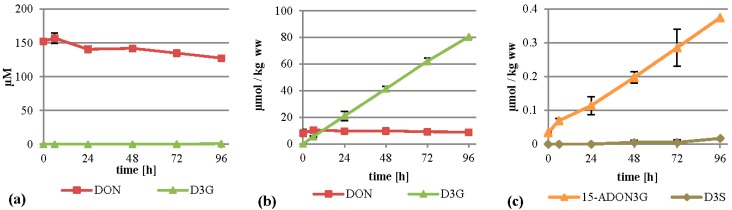
Metabolization of DON by *T. aestivum* suspension cultures. (**a**) Supernatant; (**b**) cell extract samples; (**c**) novel minor metabolites found in cell extracts. ww: wet weight.

When DON was added to the cultures to initially 75 mg/L (253 µM), the major metabolite formed was D3G. This is in concordance with a recent study by Kluger *et al.* identifying D3G as the major metabolite after inoculation of DON on wheat heads [23]. D3G was almost exclusively found in the cell extracts where it was increasing continuously and reached about 80 µmol per kilogram wet weight after 96 h (Figure 3b). The DON concentration in the cells stayed more or less constant, with values between 8.1 and 10.4 µmol per kg wet weight. The linear rise of D3G and the constant values of DON indicate that all additional DON entering the cells is metabolized into D3G. We also detected D3S in very low amounts (see Figure 3c and Table 1 for details) and DON-GSH, which was only quantified relatively due to the lack of an analytical standard. DON-GSH and processing products were also found in wheat heads after treatment with DON [23]. Furthermore, we detected 15-ADON3G in amounts up to 0.37 µmol per kg wet weight (Figure 3c), indicating that either DON or D3G is acetylated to a low extent, which is in concordance with a recently published study [23]. However, most of the DON stayed untransformed in the supernatant (Figure 3a), indicating that either DON is transported back efficiently by efflux carriers or only slowly enters the cells, leading to an about 15-fold lower intracellular concentration than in the medium.

### 2.3. Treatment with 15-ADON

**Figure 4:**
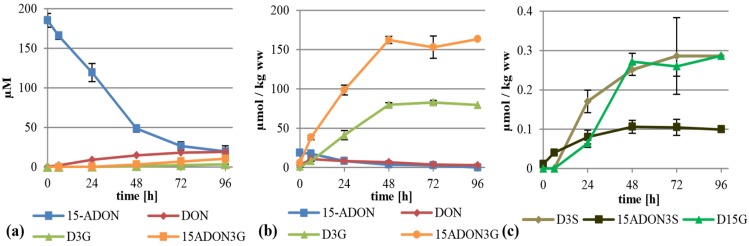
Metabolization of 15-ADON by *T. aestivum* suspension cultures. (**a**) Supernatant; (**b**) cell extract samples; (**c**) novel minor metabolites found in cell extracts.

When supplementing 15-ADON (75 mg/L, 221 µM) instead of DON we expected deacetylation as first step of metabolization, similar to the observation that only up to 20% of the total DON has been found as ADON in naturally contaminated samples [7], although 15-ADON or 3-ADON are usually the major compounds in axenic *Fusarium* cultures of the corresponding chemotype. As expected, 15-ADON was quickly metabolized, with only about 11% left in the supernatant after 96 h (Figure 4a). The major extracellular metabolite of 15-ADON was DON with about 19 µM after 96 h. Furthermore, about 10.4 µM 15-ADON3G compared to only 3.4 µM D3G were detectable in the supernatant. However, most of the metabolites were found again in the cell extracts. Interestingly, up to 163 µmol per kg wet weight 15-ADON3G in addition to 83 µmol per kg wet weight of D3G was found (Figure 4b). This clearly shows that 15-ADON3G, presumably a non-toxic compound, sterically unable to interact with the ribosomal target like D3G [27], is the major metabolization product of 15-ADON. After 6 h, there is a peak of DON in the cell extract with about 11 µmol per kg wet weight. Besides DON, 15-ADON3G and D3G, up to 17 µmol 15-ADON per kg wet weight, and the phase II metabolites D3S, 15-ADON-3-sulfate (15-ADON3S) and tentatively identified DON-15-β-d-glucoside (D15G) were present in concentrations up to 0.29, 0.11 and 0.29 µmol per kg wet weight, respectively (Figure 4b,c, Supplemental Table S1). To our knowledge, this is the first time that 15-ADON3S and D15G are described as DON metabolites in any kind of plant material. Again, DON-GSH was detected but only quantified relatively (Supplemental Table S1).

**Table 1:** Retention behavior and optimized ESI-MS and ESI-MS/MS parameters of the target analytes (extended from Warth *et al.* [24]). ESI-MS/MS was performed in positive or negative SRM mode using fast polarity switching.

Analyte	RT (min)	Q1 (*m*/*z*)	DP ^a^ (V)	Q3 ^b^ (*m*/*z*)	Relative Intensity ^c^	CE ^b,d^ (eV)
Deoxynivalenol DON	7.0	355.1 [M + Ac]^−^	−20	265.0/247.0	32%	−20/−22
3-acetyl-DON 3-ADON	8.8	397.0 [M + Ac]^−^	−50	306.9/173.0	53%	−20/−20
15-acetyl-DON 15-ADON	8.7	339.1 [M + H]^+^	91	261.0/137.2	47%	17/30
3,15-di-acetyl-DON 3,15-diADON	9.5	381.1 [M + H]^+^	71	231.0/321.1	51%	23/13
DON-3-glucoside D3G	6.7	517.0 [M + Ac]^−^	−50	457.1/427.1	24%	−18/−18
DON-15-glucoside D15G	6.5	517.0 [M + Ac]^−^	−50	457.1/59.0	9%	−18/−50
15-acetyl-DON-3-glucoside 15-ADON3G	8.4	518.0 [M + NH_4_]^+^	50	339.1/321.1	27%	15/25
DON-3-sulfate D3S	5.8	375.0 [M − H]^−^	−125	345.0/246.9	68%	−36/−82
DON-15-sulfate D15S	5.7	375.0 [M − H]^−^	−110	97.0/163.1	38%	−38/−50
15-acetyl-DON-3-sulfate 15-ADON3S	8.4	417 [M − H]^−^	−45	97.0/192.8	19%	−82/−40
DON-glutathione DON-GSH	4.0	602.0 [M − H]^−^	−40	306.1/143.0	3%	−30/−30

Note: ^a^ Declustering potential; ^b^ Values are given in the order quantifier ion/qualifier ion; ^c^ Signal intensity of the qualifier transition in relation to the quantifier (qualifier/quantifier × 100); ^d^ Collision energy.

### 2.4. Treatment with 3-ADON

**Figure 5:**
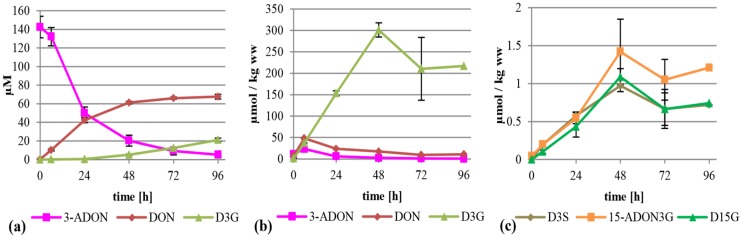
Metabolization of 3-ADON by *T. aestivum* suspension cultures. (**a**) Supernatant; (**b**) cell extract samples; (**c**) novel minor metabolites in cell extract.

At first glance, a similar picture can be seen when 3-ADON instead of 15-ADON was added to the suspension culture (75 mg/L, 221 µM). 3-ADON was deacetylated and metabolized faster compared to 15-ADON into DON, D3G and other metabolites, with only 4% of the initially present 3-ADON left in the supernatant after 96 h. The major metabolites in the supernatant were DON and D3G at about 68 and 21 µM (Figure 5a). The high DON value could indicate that either extracellular enzymes were deacetylating 3-ADON, or that 3-ADON is intracellularly deacetylated and efficiently transported out of the cells. The intracellular DON is obviously efficiently converted into D3G. Up to 301 µmol per kg wet weight was found in the cells (Figure 5b), corresponding to an about 1.36 fold accumulation. Intracellular DON values peaked with about 48.4 µmol per kg wet weight after six hours, which is more than four times higher than after all other treatments. The difference is still significant if the sum of all intracellular toxic metabolites (15-ADON and DON) after six hours is compared, being 48.4, 28.2, 10.4 and 10.9 µmol per kg wet weight after 3-ADON, 15-ADON, DON and 3,15-diADON treatment, respectively. Additionally, about 23 µmol per kg wet weight of 3-ADON was detected. Furthermore minor amounts of 15-ADON3G, D3S and D15G were found predominantly in the cell extracts but also in the supernatant (Figure 5c, Supplemental Table S1). Again, DON-GSH was detected but only quantified relatively (Supplemental Table S1). A potential 3-ADON-15-glucoside or 3-ADON-15-sulfate was not detected.

### 2.5. Treatment with 3,15-diADON

**Figure 6:**
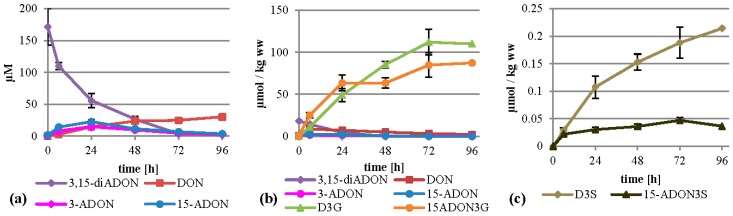
Metabolization of 3,15-diADON by *T. aestivum* suspension cultures. (**a**) Supernatant; (**b**) cell extract samples; (**c**) novel minor metabolites in cell extract.

Metabolization of 3,15-diADON (75 mg/L, 197 µM) showed an intermediate behavior between 3-ADON and 15-ADON. It was deacetylated fast and almost completely within 96 h, more similar to 3-ADON treatment, but similar to 15-ADON treatment no high levels of DON arose in the supernatant (Figure 6a). Additionally, small amounts of 3-ADON and 15-ADON are temporally detectable in the supernatant, peaking after 24 h. It is unclear whether 3,15-diADON is partly hydrolyzed by a extracellular esterase, or whether intracellularly generated products are transported back into the medium. Inside the cells, high amounts of D3G and 15-ADON3G were formed summing up to about 112 and 87 µmol per kg wet weight (Figure 6b). DON, 3-ADON and 15-ADON values peaked after six hours with 8.6, 1.7 and 2.2 µmol per kg wet weight, respectively. 3,15-diADON was highest at the starting point with about 18 µmol per kg wet weight, showing that 3,15-diADON effectively enters the cells and gets subsequently metabolized. Furthermore, we found D3S and 15‑ADON3S in amounts up to 0.21 µmol per kg wet weight (Figure 6c). DON-GSH was detected but only quantified relatively (Supplemental Table S1).

### 2.6. Occurrence of the Novel Plant Conjugates DON-15-Glucoside and 15-ADON-3-Sulfate

In addition to previously described DON-conjugates, we were able to detect and identify D15G tentatively and 15-ADON3S unambiguously in the cell extracts using our tailored LC-MS/MS method in low amounts (see Section 2.2, Section 2.3, Section 2.4 and Section 2.5 and Supplemental Table S1).

**Figure 7:**
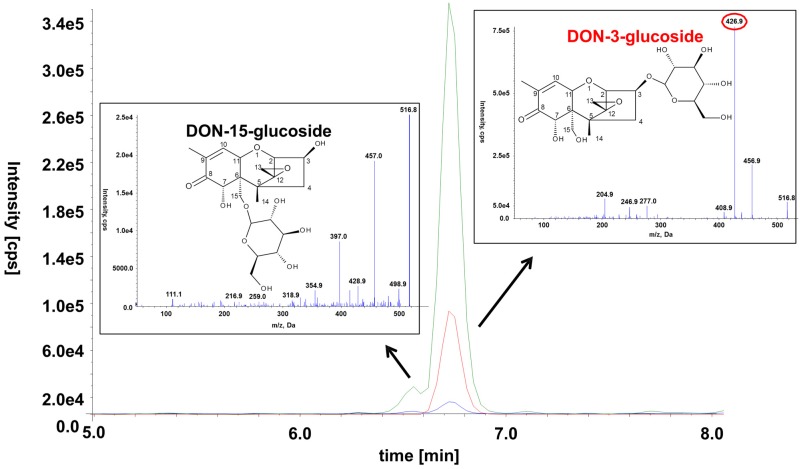
Chemical structure, SRM chromatograms and MS/MS spectra of the two DON-glucoside isomers in a diluted wheat sample. The following transitions are displayed: *m*/*z* 517.1→457.1 (green), *m*/*z* 517.1→427.1 (red), *m*/*z* 517.1→59.0 (blue). The transition *m*/*z* 517.1→427.1 is specific to D3G and can thus be used to distinguish between the two isomers. The MS/MS spectra of the precursor ion at *m*/*z* 517.0 [M + Ac]^−^ were recorded at a collision energy of −20 eV.

D15G was chemically synthetized before [28] but never detected in any naturally contaminated or artificially infected sample. We assume that this is mainly caused by the very similar chemical properties of its isomer D3G, which is much more abundant and probably co-elutes on most reversed phase columns under typically used chromatographic conditions. Here, we were able to separate the two isomers using a special stationary phase, which was optimized for the retention of polar molecules, and an optimized very flat gradient [24]. Another possible reason for D15G not having been described earlier might be that older mass spectrometers were not sensitive enough to allow the detection of this conjugate. After 3-ADON and 15-ADON treatment, D15G was tentatively identified via LC-MS/MS in the cell extract as well as in the supernatant. Loss of a H_2_CO moiety from the -CH_2_OH group attached to the C-6 position of the D3G resulted in an intense peak at *m/z* 427 which is absent in the case of D15G. It would also be possible that the detected compound is DON-7-Glc, however, then also the loss of the H_2_CO should be visible in the MS/MS spectrum (Figure 7). This principle was already described earlier for the differentiation of DON‑conjugate isomers [24,28,29]. To estimate the relative importance of D15G in comparison to D3G, we quantified it using the calibration curve of D3G and the transition *m*/*z* 517.1→457.1. It should be noted that this does not allow for an accurate quantification as the mass spectrometric response might be different. 15-ADON3S was identified based on comparison of the retention time and the MS/MS spectra of a recently chemically synthetized and NMR confirmed reference standard [30].

3-ADON-15-sulfate and DON-3,15-di-sulfate, for which also authentic standards were available, were both included in the analytical method but not detected in any sample.

## 3. Materials and Methods

### 3.1. Chemicals and Reagents

Acetonitrile (ACN, LC gradient grade) was purchased from VWR (Leuven, Belgium). Ammonium acetate (MS grade), Tris(hydroxymethyl)-aminomethan (purity ≥99.9%) and Uridine 5′-diphosphoglucose disodium salt hydrate from *Saccharomyces cerevisiae* (purity ≥98%) from Sigma-Aldrich (Schnelldorf, Germany). Water was purified by an Elga Purelab ultra analytic system (Veolia Water, Buckinghamshire, UK). D3S, D15S, 3ADON15S, 15-ADON3S and DON-3,15-disulfate were all synthesized using a sulfuryl imidazolium salt as described by Fruhmann *et al.* [30]. D3G, 3-ADON, 15-ADON and DON for analytical purpose were purchased from Romer Labs Diagnostic GmbH (Tulln, Austria) whereas all toxins for the treatment of cells were prepared as described previously [30].

### 3.2. Wheat Suspension Culture Assays

For the suspension culture assay, dense *T. aestivum* L. emend. Fiori and Paol. cv. Heines Koga II (PC-998) cultures obtained from DSMZ (Braunschweig, Germany) cultivated at 20 °C in light were re-suspended in about 83% of the original volume in fresh B5 medium (194 ± 34.5 mg wet weight per mL) and spiked with 75 mg/L 3-ADON, 15-ADON, 3,15-diADON or DON. Samples of 1 mL were taken, utilizing cut 5 mL tips, after “0” (about 15 min manipulation time), 6, 24, 48, 72, 96 h post incubation (100 rpm, 21 °C in light) and transferred into 1.5 mL safe lock microcentrifuge tubes. The supernatant and cell extracts were analyzed separately. The samples were centrifuged for 5 min at 4000 × g. The supernatant was removed and diluted to 50% ethanol (final) and stored for analysis at −20 °C. The pellet was washed once with medium diluted to 20% ethanol, centrifuged again, and then the medium was removed by aspiration. After determination of the wet weight, 250 µL ethanol per 100 mg wet weight and a steel ball was added to the pellet and ground in a retch mill at 25 Hz for two min. After centrifuging for 5 min at 20,000 × g the supernatant was diluted to 50% ethanol and stored at −20 °C until analysis. As controls, untreated suspension cultures and empty medium containing 3-ADON or 15-ADON were monitored. The concentrations of the toxins and metabolization products were determined using an LC-MS/MS system as described in Section 3.3. The metabolization of each toxin was assayed in three biological replicates.

### 3.3. Liquid Chromatography-Tandem Mass Spectrometry Analysis

An extension of the method published by Warth *et al.* [24] was utilized for chromatographic separation, detection, and quantification of DON and its metabolites. In brief, a QTRAP 6500 system (AB Sciex, Foster City, CA, USA) equipped with an IonDrive™ Turbo V electrospray ionization (ESI) source and interfaced with an Agilent 1290 series UHPLC system (Waldbronn, Germany) was applied. Gradient elution at 30 °C was performed within 14 min on an Atlantis T3 column (3.0 × 150 mm; Waters, Wexford, Ireland) with 3 μm particle size and a C18 pre-column (Gemini® 4 × 3 mm i.d.; Phenomenex, Torrance, CA, USA) operated at a flow rate of 600 μL/min. Eluent A (water) and eluent B (ACN) contained both 20 mM ammonium acetate. After an initial time period of 0.5 min at 95% eluent A, the percentage of eluent B was linearly raised to 15% until 6 min. Then, eluent B was raised to 100% until 9 min followed by a holding time of 2 min and subsequent 3 min of column re-equilibration at 95% eluent A.

ESI-MS/MS was performed in positive or negative SRM mode using fast polarity switching for all analytes investigated in this study. Two individual transitions were monitored for each analyte. All measurements were done with the following settings: low mass range, source temperature (550°C), curtain gas (30 psi; 69 kPa of max. 99.5% nitrogen), ion source gas 1 (sheath gas, 60 psi), ion source gas 2 (drying gas, 60 psi), and collision gas (nitrogen, high). The ion spray voltage was set to −4000 V.

Analyte-dependent MS/MS parameters were optimized via direct infusion of reference standards if available (Table 1). MS/MS spectra (enhanced product ion scans) were recorded at a collision energy of −20 eV and a scan rate of 1000 Da/s. For external calibration, curves (1/*x* weighted) were generated and for instrumental control and data evaluation, Analyst software (version 1.6.2; AB Sciex) was applied.

### 3.4. Enzymatic Glucosylation of 15-ADON to 15-ADON-3-O-Glucoside

The reaction pre-mixture contained 200 mM Tris-HCl pH 7, 2 mM UDP-glucose and 10 mg/L 15-ADON. To 100 µL of this solution, 100 µL of affinity-purified recombinant UDP-glucosyltransferase from rice (NM_001058779.1) [26] was added (3.4 mg/mL protein) and incubated for two hours at room temperature. Afterwards, 200 µL acetonitrile was added and immediately measured with LC-MS/MS.

## 4. Conclusions

**Scheme 1:**
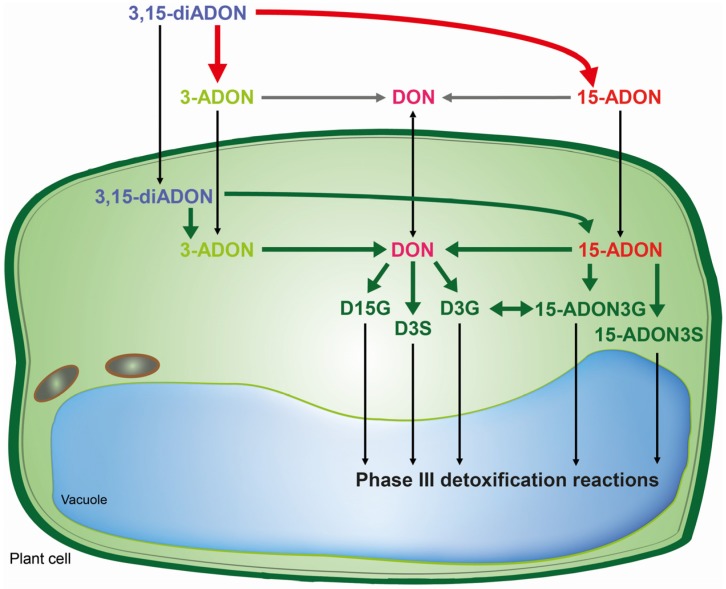
Metabolic fate of DON and its acetylated derivatives in wheat. Bold arrows indicate enzymatic catalysis by *F. graminearum* (red) or wheat (green). Thin black arrows indicate a translocation and grey arrows unknown steps. The 3,15-diADON becomes deacetylated either to 15-ADON or to 3-ADON depending on the chemotype of the strain by the *F. graminearum* Tri8 esterase.

This is the first report on the presence of the masked mycotoxins D15G, 15-ADON3G and 15-ADON3S in toxin-treated wheat suspension cultures. The *in vivo* assays shed new light on biotransformation of DON and its acetylated derivatives, and identified different patterns of metabolization depending on the toxin added. Wheat is capable of rapidly hydrolyzing acetylated DON derivatives. Obviously, DON offers the most options due to the possibility for conjugation with glucose and sulfate to both the C3-OH and C15-OH. However, for 15-ADON the formation of 15-ADON3G seems to be the major detoxification pathway and to a limited extent 15-ADON3S is formed as well. The potential 3-ADON counterparts 3-ADON-15-glucoside and 3-ADON-15-sulfate as well as DON-3,15-disulfate, were not detected in our study. It seems that the major difference between metabolization of 3- and 15-ADON is that 15-ADON can be directly conjugated into the compound 15-ADON3G, which is rather unstable, and into 15-ADON3S which is not possible for 3-ADON since the acetyl group blocks the C3-OH. Wheat suspension cultures seem to cope better with 15-ADON and DON than with the presumed non-toxic 3-ADON. In Scheme 1, we summarized the pathways concerning the metabolic fate of DON and its acetylated derivatives in wheat. In the future, we aim to investigate the implications of these results concerning population shifts in *Fusarium graminearum* chemotype composition in more detail.

## Supplementary Materials

Supplementary materials can be accessed at: http://www.mdpi.com/2072-6651/7/8/3112/s1.
