# Supplementary material for: The Metabolic Fate of Deoxynivalenol and Its Acetylated Derivatives in a Wheat Suspension Culture: Identification and Detection of DON-15-O-Glucoside, 15-Acetyl-DON-3-O-Glucoside and 15-Acetyl-DON-3-Sulfate
Source: Toxins (Basel). 2015 Aug 12;7(8):3112–26. doi: 10.3390/toxins7083112 (PMC4549741; doi:10.3390/toxins7083112)
Supplement: Supplementary file 1 [file toxins-07-03112-s001.pdf]

Supplementary Materials

Table S1. All individual samples of wheat suspension culture assay according to section 3.2. LOD: limit of detection, ww: wet weight.

Sample Type	Replicate	Time (h)	Toxin Added	DON (µM)	D3G (µM)	D3S (µM)	D15S (µM)	3-ADON (µM)	15-ADON (µM)	15-ADON3G (µM)	15-ADON3S (µM)	D15G (µM)	3,15-diADON (µM)	DON-GSH (Peak Area)
supernatant	1	0	3-ADON	0.31	<LOD	<LOD	<LOD	126	<LOD	<LOD	<LOD	<LOD	-	<LOD
supernatant	2	0	3-ADON	0.31	<LOD	<LOD	<LOD	148	<LOD	<LOD	<LOD	<LOD	-	<LOD
supernatant	3	0	3-ADON	0.33	<LOD	<LOD	<LOD	153	<LOD	<LOD	<LOD	<LOD	-	<LOD
supernatant	1	6	3-ADON	11.2	0.21	<LOD	<LOD	132	<LOD	<LOD	<LOD	<LOD	-	<LOD
supernatant	2	6	3-ADON	8.63	0.21	<LOD	<LOD	144	<LOD	<LOD	<LOD	<LOD	-	<LOD
supernatant	3	6	3-ADON	10.9	0.22	<LOD	<LOD	120	<LOD	<LOD	<LOD	<LOD	-	<LOD
supernatant	1	24	3-ADON	43.5	0.54	<LOD	<LOD	46.2	<LOD	<LOD	<LOD	<LOD	-	2551
supernatant	2	24	3-ADON	38.7	0.47	<LOD	<LOD	59.2	<LOD	<LOD	<LOD	<LOD	-	2251
supernatant	3	24	3-ADON	44.8	0.59	<LOD	<LOD	44.3	<LOD	<LOD	<LOD	<LOD	-	2176
supernatant	1	48	3-ADON	61.3	5.24	<LOD	<LOD	15.5	<LOD	0.02	<LOD	<LOD	-	13430
supernatant	2	48	3-ADON	59.5	4.40	<LOD	<LOD	28.4	<LOD	0.02	<LOD	<LOD	-	11860
supernatant	3	48	3-ADON	63.0	5.86	<LOD	<LOD	16.9	<LOD	0.02	<LOD	<LOD	-	2926
supernatant	1	72	3-ADON	65.2	12.6	0.05	<LOD	6.75	<LOD	0.06	<LOD	0.04	-	14030
supernatant	2	72	3-ADON	67.7	12.5	0.05	<LOD	15.2	<LOD	0.05	<LOD	0.03	-	15080
supernatant	3	72	3-ADON	65.1	12.2	0.05	<LOD	5.77	<LOD	0.04	<LOD	0.04	-	12460
supernatant	1	96	3-ADON	67.8	20.1	0.08	<LOD	3.25	<LOD	0.09	<LOD	0.10	-	21460
supernatant	2	96	3-ADON	70.6	24.0	0.02	<LOD	10.0	<LOD	0.13	<LOD	<LOD	-	-
supernatant	3	96	3-ADON	64.5	19.6	0.02	<LOD	2.41	<LOD	0.11	<LOD	<LOD	-	-
supernatant	1	0	15-ADON	0.44	<LOD	<LOD	<LOD	10.9	195	0.00	<LOD	<LOD	-	150.1
supernatant	2	0	15-ADON	0.48	<LOD	<LOD	<LOD	12.0	187	0.00	<LOD	<LOD	-	75.05
supernatant	3	0	15-ADON	0.42	<LOD	<LOD	<LOD	11.3	174	0.00	<LOD	<LOD	-	150.1
supernatant	1	6	15-ADON	2.17	<LOD	<LOD	<LOD	9.76	170	0.03	<LOD	<LOD	-	225.1
supernatant	2	6	15-ADON	1.90	<LOD	<LOD	<LOD	9.82	168	0.02	<LOD	<LOD	-	<LOD
supernatant	3	6	15-ADON	1.96	<LOD	<LOD	<LOD	10.7	160	0.02	<LOD	<LOD	-	<LOD
supernatant	1	24	15-ADON	9.68	0.29	<LOD	<LOD	5.48	104	0.32	0.00	<LOD	-	<LOD

Table S1. *Cont.*

Sample Type	Replicate	Time (h)	Toxin Added	DON (µM)	D3G (µM)	D3S (µM)	D15S (µM)	3-ADON (µM)	15-ADON (µM)	15-ADON3G (µM)	15-ADON3S (µM)	D15G (µM)	3,15-diADON (µM)	DON-GSH (Peak Area)
supernatant	2	24	15-ADON	8.94	0.26	<LOD	<LOD	6.41	123	0.23	0.00	<LOD	-	825.4
supernatant	3	24	15-ADON	8.64	0.25	<LOD	<LOD	7.65	131	0.22	0.00	<LOD	-	600.3
supernatant	1	48	15-ADON	14.9	1.12	<LOD	<LOD	3.15	44.2	3.26	0.01	<LOD	-	2101
supernatant	2	48	15-ADON	14.9	1.04	<LOD	<LOD	3.76	47.1	3.28	0.00	<LOD	-	2776
supernatant	3	48	15-ADON	14.6	0.95	<LOD	<LOD	3.19	54.9	2.61	0.00	<LOD	-	11330
supernatant	1	72	15-ADON	17.8	2.30	<LOD	<LOD	1.80	19.5	7.06	0.01	0.01	-	3827
supernatant	2	72	15-ADON	18.1	2.12	<LOD	<LOD	2.09	27.7	6.40	0.01	<LOD	-	2701
supernatant	3	72	15-ADON	18.7	2.33	<LOD	<LOD	2.81	32.2	7.15	0.01	<LOD	-	2626
supernatant	1	96	15-ADON	18.3	3.74	<LOD	<LOD	1.06	11.7	10.1	0.01	0.02	-	4427
supernatant	2	96	15-ADON	18.7	3.09	<LOD	<LOD	0.80	18.7	9.82	<LOD	<LOD	-	-
supernatant	3	96	15-ADON	20.2	3.51	<LOD	<LOD	1.28	28.9	11.4	<LOD	<LOD	-	-
supernatant	1	0	3,15-diADON	0.08	<LOD	<LOD	<LOD	0.57	0.79	<LOD	<LOD	<LOD	188	<LOD
supernatant	2	0	3,15-diADON	0.12	<LOD	<LOD	<LOD	0.54	2.73	<LOD	<LOD	<LOD	195	<LOD
supernatant	3	0	3,15-diADON	0.05	<LOD	<LOD	<LOD	0.47	1.04	<LOD	<LOD	<LOD	131	<LOD
supernatant	1	6	3,15-diADON	2.18	<LOD	<LOD	<LOD	6.97	12.1	<LOD	<LOD	<LOD	105	<LOD
supernatant	2	6	3,15-diADON	2.21	<LOD	<LOD	<LOD	7.29	13.0	<LOD	<LOD	<LOD	117	<LOD
supernatant	3	6	3,15-diADON	3.06	<LOD	<LOD	<LOD	8.63	17.0	<LOD	<LOD	<LOD	108	<LOD
supernatant	1	24	3,15-diADON	14.7	0.09	<LOD	<LOD	14.9	21.5	0.09	<LOD	<LOD	54.0	<LOD
supernatant	2	24	3,15-diADON	14.6	0.08	<LOD	<LOD	16.0	26.7	0.11	<LOD	<LOD	70.2	<LOD
supernatant	3	24	3,15-diADON	14.6	0.08	<LOD	<LOD	13.0	19.4	0.09	<LOD	<LOD	43.2	157.1
supernatant	1	48	3,15-diADON	24.0	0.81	<LOD	<LOD	10.4	15.5	1.00	<LOD	<LOD	16.2	391.9
supernatant	2	48	3,15-diADON	22.5	0.70	<LOD	<LOD	11.1	16.6	1.01	<LOD	<LOD	20.1	471.4
supernatant	3	48	3,15-diADON	23.7	0.65	<LOD	<LOD	7.94	11.7	0.84	<LOD	<LOD	9.66	<LOD
supernatant	1	72	3,15-diADON	24.5	1.51	<LOD	<LOD	4.79	6.17	1.84	<LOD	<LOD	3.68	<LOD
supernatant	2	72	3,15-diADON	23.2	1.48	<LOD	<LOD	5.61	8.17	1.96	<LOD	<LOD	4.40	<LOD

Table S1. *Cont.*

Sample Type	Replicate	Time (h)	Toxin Added	DON (µM)	D3G (µM)	D3S (µM)	D15S (µM)	3-ADON (µM)	15-ADON (µM)	15-ADON3G (µM)	15-ADON3S (µM)	D15G (µM)	3,15-diADON (µM)	DON-GSH (Peak Area)
supernatant	3	72	3,15-diADON	26.4	1.45	<LOD	<LOD	3.47	5.78	1.66	<LOD	<LOD	1.74	471.1
supernatant	1	96	3,15-diADON	30.1	3.06	<LOD	<LOD	2.72	3.47	3.51	<LOD	<LOD	1.09	785.4
supernatant	1	0	DON	153	<LOD	<LOD	<LOD	<LOD	<LOD	<LOD	<LOD	<LOD	<LOD	<LOD
supernatant	2	0	DON	151	<LOD	<LOD	<LOD	<LOD	<LOD	<LOD	<LOD	<LOD	<LOD	<LOD
supernatant	3	0	DON	153	<LOD	<LOD	<LOD	<LOD	<LOD	<LOD	<LOD	<LOD	<LOD	<LOD
supernatant	1	6	DON	153	<LOD	<LOD	<LOD	<LOD	<LOD	<LOD	<LOD	<LOD	<LOD	<LOD
supernatant	2	6	DON	168	<LOD	<LOD	<LOD	<LOD	<LOD	<LOD	<LOD	<LOD	<LOD	<LOD
supernatant	3	6	DON	151	<LOD	<LOD	<LOD	<LOD	<LOD	<LOD	<LOD	<LOD	<LOD	<LOD
supernatant	1	24	DON	145	<LOD	<LOD	<LOD	<LOD	<LOD	<LOD	<LOD	<LOD	<LOD	<LOD
supernatant	2	24	DON	142	<LOD	<LOD	<LOD	<LOD	<LOD	<LOD	<LOD	<LOD	<LOD	<LOD
supernatant	3	24	DON	135	<LOD	<LOD	<LOD	<LOD	<LOD	<LOD	<LOD	<LOD	<LOD	1658
supernatant	1	48	DON	141	0.14	<LOD	<LOD	<LOD	<LOD	<LOD	<LOD	<LOD	<LOD	1963
supernatant	2	48	DON	145	0.18	<LOD	<LOD	<LOD	<LOD	<LOD	<LOD	<LOD	<LOD	1885
supernatant	3	48	DON	139	0.17	<LOD	<LOD	<LOD	<LOD	<LOD	<LOD	<LOD	<LOD	4555
supernatant	1	72	DON	137	0.44	<LOD	<LOD	<LOD	<LOD	<LOD	<LOD	<LOD	<LOD	6283
supernatant	2	72	DON	138	0.40	<LOD	<LOD	<LOD	<LOD	<LOD	<LOD	<LOD	<LOD	4241
supernatant	3	72	DON	131	0.47	<LOD	<LOD	<LOD	<LOD	<LOD	<LOD	<LOD	<LOD	2513
supernatant	1	96	DON	128	0.87	<LOD	<LOD	<LOD	<LOD	<LOD	<LOD	<LOD	<LOD	3377

Table S1. *Cont.*

Sample Type	Replicate	Time (h)	Toxin Added	DON (µmol/ kg ww)	D3G (µmol/ kg ww)	D3S (µmol/ kg ww)	D15S (µmol/ kg ww)	3-ADON (µmol/ kg ww)	15-ADON (µmol/ kg ww)	15-ADON3G (µmol/ kg ww)	15-ADON3S (µmol/ kg ww)	D15G (µmol/ kg ww)	3,15-diADON (µmol/ kg ww)	DON-GSH (Peak Area)
extract	1	0	3-ADON	8.38	1.44	<LOD	<LOD	11.6	<LOD	0.05	<LOD	<LOD	-	2701
extract	2	0	3-ADON	6.63	1.16	<LOD	<LOD	11.2	<LOD	0.05	<LOD	<LOD	-	1726
extract	3	0	3-ADON	6.01	1.24	<LOD	<LOD	11.3	<LOD	0.04	<LOD	<LOD	-	1951
extract	1	6	3-ADON	48.3	34.9	0.20	<LOD	23.8	<LOD	0.19	<LOD	0.13	-	118800
extract	2	6	3-ADON	42.8	35.6	0.18	<LOD	20.4	<LOD	0.17	<LOD	0.10	-	120700
extract	3	6	3-ADON	54.0	41.8	0.21	<LOD	26.2	<LOD	0.23	<LOD	0.08	-	165900
extract	1	24	3-ADON	25.8	150	0.56	<LOD	5.78	<LOD	0.58	<LOD	0.61	-	331900
extract	2	24	3-ADON	25.7	149	0.53	<LOD	8.28	<LOD	0.45	<LOD	0.41	-	385700
extract	3	24	3-ADON	19.6	161	0.65	<LOD	4.77	<LOD	0.61	<LOD	0.28	-	327700
extract	1	48	3-ADON	17.7	296	0.92	<LOD	2.07	<LOD	1.20	<LOD	1.24	-	372600
extract	2	48	3-ADON	17.9	324	0.92	<LOD	3.13	<LOD	1.04	<LOD	1.00	-	391300
extract	3	48	3-ADON	16.8	284	1.08	<LOD	2.31	<LOD	2.02	<LOD	1.03	-	124100
extract	1	72	3-ADON	7.38	137	0.42	<LOD	0.59	<LOD	0.75	<LOD	0.47	-	146600
extract	2	72	3-ADON	12.8	311	1.02	<LOD	1.88	<LOD	1.40	<LOD	0.96	-	349000
extract	3	72	3-ADON	8.34	184	0.56	<LOD	0.57	<LOD	0.99	<LOD	0.56	-	173900
extract	1	96	3-ADON	10.9	217	0.72	<LOD	0.54	<LOD	1.21	<LOD	0.74	-	171000
extract	1	0	15-ADON	2.78	0.97	<LOD	<LOD	1.39	20.0	6.97	0.02	<LOD	-	900.5
extract	2	0	15-ADON	3.23	1.00	<LOD	<LOD	1.92	23.0	6.36	0.01	<LOD	-	1201
extract	3	0	15-ADON	2.21	0.79	<LOD	<LOD	1.19	13.1	5.04	0.01	<LOD	-	975.4
extract	1	6	15-ADON	10.6	7.27	<LOD	<LOD	1.60	16.6	35.6	0.04	<LOD	-	26410
extract	2	6	15-ADON	10.0	7.69	<LOD	<LOD	1.46	18.6	36.8	0.04	0.07	-	18830
extract	3	6	15-ADON	11.6	9.10	<LOD	<LOD	1.55	17.3	42.3	0.05	0.07	-	26940
extract	1	24	15-ADON	8.69	43.4	0.21	<LOD	0.80	9.31	107	0.09	0.05	-	102000
extract	2	24	15-ADON	8.80	46.8	0.15	<LOD	0.70	8.19	97.3	0.10	0.30	-	98670
extract	3	24	15-ADON	7.34	33.1	0.15	<LOD	0.58	7.01	91.7	0.06	0.26	-	100900
extract	1	48	15-ADON	7.62	79.2	0.27	<LOD	0.31	4.76	167	0.12	0.26	-	126500

Table S1. *Cont.*

Sample Type	Replicate	Time (h)	Toxin Added	DON (µmol/ kg ww)	D3G (µmol/ kg ww)	D3S (µmol/ kg ww)	D15S (µmol/ kg ww)	3-ADON (µmol/ kg ww)	15-ADON (µmol/ kg ww)	15-ADON3G (µmol/ kg ww)	15-ADON3S (µmol/ kg ww)	D15G (µmol/ kg ww)	3,15-diADON (µmol/ kg ww)	DON-GSH (Peak Area)
extract	2	48	15-ADON	6.55	76.6	0.24	<LOD	0.30	5.79	156	0.08	0.26	-	120100
extract	3	48	15-ADON	6.17	83.4	0.24	<LOD	0.21	0.00	163	0.11	0.23	-	465300
extract	1	72	15-ADON	3.88	85.3	0.42	<LOD	0.12	4.41	173	0.13	0.29	-	94650
extract	2	72	15-ADON	3.50	84.0	0.24	<LOD	0.12	2.02	146	0.09	0.29	-	95280
extract	3	72	15-ADON	4.08	79.0	0.20	<LOD	0.17	1.52	141	0.09	0.00	-	81280
extract	1	96	15-ADON	2.92	79.5	0.29	<LOD	0.10	0.00	163	0.10	0.00	-	55260
extract	1	0	3,15-diADON	0.48	0.14	<LOD	<LOD	0.84	1.17	0.95	<LOD	<LOD	17.6	314.1
extract	2	0	3,15-diADON	0.54	0.16	<LOD	<LOD	0.83	1.24	0.96	<LOD	<LOD	19.4	<LOD
extract	3	0	3,15-diADON	0.48	0.11	<LOD	<LOD	0.78	1.45	0.87	<LOD	<LOD	18.0	<LOD
extract	1	6	3,15-diADON	8.15	10.9	0.03	<LOD	1.51	2.48	26.7	0.02	<LOD	12.5	43900
extract	2	6	3,15-diADON	8.47	9.43	0.02	<LOD	1.71	2.39	20.6	0.02	<LOD	16.1	29980
extract	3	6	3,15-diADON	9.32	13.0	0.03	<LOD	1.84	1.76	27.3	0.02	<LOD	14.8	41160
extract	1	24	3,15-diADON	8.01	39.0	0.08	<LOD	1.25	1.54	54.2	0.03	<LOD	3.73	110200
extract	2	24	3,15-diADON	7.46	57.1	0.13	<LOD	1.65	2.67	76.0	0.04	<LOD	5.98	141500
extract	3	24	3,15-diADON	6.52	51.4	0.11	<LOD	1.22	2.51	59.3	0.03	<LOD	4.29	92670
extract	1	48	3,15-diADON	5.79	80.0	0.17	<LOD	0.85	1.34	76.0	0.03	<LOD	<LOD	106200
extract	2	48	3,15-diADON	5.43	89.5	0.14	<LOD	1.05	1.01	87.4	0.04	<LOD	<LOD	110100
extract	3	48	3,15-diADON	4.18	86.9	0.14	<LOD	0.65	0.86	90.0	0.03	<LOD	<LOD	146400
extract	1	72	3,15-diADON	3.73	105	0.17	<LOD	0.47	0.41	95.2	0.05	<LOD	<LOD	97860
extract	2	72	3,15-diADON	4.04	133	0.23	<LOD	0.56	<LOD	118	0.05	<LOD	<LOD	153600
extract	3	72	3,15-diADON	2.54	97.9	0.16	<LOD	0.27	<LOD	84.0	0.04	<LOD	<LOD	95720
extract	1	96	3,15-diADON	2.37	110	0.21	<LOD	0.21	<LOD	86.6	0.04	<LOD	<LOD	70180
extract	1	0	DON	11.5	<LOD	<LOD	<LOD	<LOD	<LOD	0.03	<LOD	<LOD	<LOD	3535
extract	2	0	DON	7.62	<LOD	<LOD	<LOD	<LOD	<LOD	0.04	<LOD	<LOD	<LOD	1334
extract	3	0	DON	5.15	<LOD	<LOD	<LOD	<LOD	<LOD	0.03	<LOD	<LOD	<LOD	942.5
extract	1	6	DON	11.6	6.24	<LOD	<LOD	<LOD	<LOD	0.07	<LOD	<LOD	<LOD	76.07

Table S1. *Cont.*

Sample Type	Replicate	Time (h)	Toxin Added	DON (µmol/ kg ww)	D3G (µmol/ kg ww)	D3S (µmol/ kg ww)	D15S (µmol/ kg ww)	3-ADON (µmol/ kg ww)	15-ADON (µmol/ kg ww)	15-ADON3G (µmol/ kg ww)	15-ADON3S (µmol/ kg ww)	D15G (µmol/ kg ww)	3,15-diADON (µmol/ kg ww)	DON-GSH (Peak Area)
extract	2	6	DON	9.30	4.66	<LOD	<LOD	<LOD	<LOD	0.06	<LOD	<LOD	<LOD	<LOD
extract	3	6	DON	10.2	5.57	<LOD	<LOD	<LOD	<LOD	0.08	<LOD	<LOD	<LOD	69460
extract	1	24	DON	9.84	22.0	<LOD	<LOD	<LOD	<LOD	0.14	<LOD	<LOD	<LOD	89080
extract	2	24	DON	7.71	16.0	<LOD	<LOD	<LOD	<LOD	0.08	<LOD	<LOD	<LOD	84610
extract	3	24	DON	11.2	24.4	<LOD	<LOD	<LOD	<LOD	0.12	<LOD	<LOD	<LOD	170500
extract	1	48	DON	9.52	40.0	<LOD	<LOD	<LOD	<LOD	0.21	<LOD	<LOD	<LOD	174700
extract	2	48	DON	9.28	39.8	<LOD	<LOD	<LOD	<LOD	0.17	<LOD	<LOD	<LOD	114500
extract	3	48	DON	10.4	44.0	0.02	<LOD	<LOD	<LOD	0.20	<LOD	<LOD	<LOD	131800
extract	1	72	DON	9.12	64.9	<LOD	<LOD	<LOD	<LOD	0.36	<LOD	<LOD	<LOD	147400
extract	2	72	DON	10.1	62.1	<LOD	<LOD	<LOD	<LOD	0.26	<LOD	<LOD	<LOD	131400
extract	3	72	DON	7.94	59.2	0.02	<LOD	<LOD	<LOD	0.24	<LOD	<LOD	<LOD	132100
extract	1	96	DON	8.82	80.5	0.02	<LOD	<LOD	<LOD	0.37	<LOD	<LOD	<LOD	121500
